# *Vacciniumchaozhouense*﻿ (Ericaceae), a new species from East Guangdong, China

**DOI:** 10.3897/phytokeys.236.108732

**Published:** 2023-12-08

**Authors:** Yi-Hua Tong, Pei-Zhao Ye, Jian-Hong Ding, Wei-Chao Huang, Wei Huang, Jing-Bo Ni

**Affiliations:** 1 State Key Laboratory of Plant Diversity and Specialty Crops & Key Laboratory of Plant Resources Conservation and Sustainable Utilization, South China Botanical Garden, Chinese Academy of Sciences, Guangzhou, 510650, China South China Botanical Garden, Chinese Academy of Sciences Guangzhou China; 2 South China National Botanical Garden, Guangzhou, 510650, China South China National Botanical Garden Guangzhou China; 3 Chao’an Fenghuangshan Provincial Nature Reserve, Chaozhou, Guangdong, 515655, China Chao’an Fenghuangshan Provincial Nature Reserve Chaozhou China

**Keywords:** Endangered species, morphology, taxonomy, Vaccinieae, *Vaccinium* sect. *Bracteata*

## Abstract

*Vacciniumchaozhouense* (Ericaceae), a new species from East Guangdong Province, China is described and illustrated. This new species is morphologically similar to *V.wrightii* by having flowers with persistent and leaf-like bracts, long pedicels, and white spherical-urceolate corollas, but is distinguished by having glandular trichomes on the abaxial surface of the leaf blade, shorter pedicels, sparsely pilose corolla ridges, and anther thecae longer than the tubules. A key to the new species and morphologically similar species is also provided.

## ﻿Introduction

With 470 accepted species, *Vaccinium* L. is the largest genus of the subfamily Vaccinioideae Rchb. (Ericaceae) (POWO 2023). In the account of Flora of China, 92 *Vaccinium* species were recorded (Fang and Stevens 2005). As several new species have been described in recent years, the number of *Vaccinium* species has now reached 100 for this country (Tong and Xia 2015; Tong et al. 2018, 2020, 2021a, b, 2022; Huang et al. 2022; Qin et al. 2023). Some preliminary molecular phylogenetic studies showed that *Vaccinium* is polyphyletic, with many sections considered more distinct than some genera (Stevens 1985; Kron et al. 2002; Predraza-Peñalosa and Luteyn 2011; Argent 2019). However, a comprehensive sampling is not easy for such a cosmopolitan genus, as it will need full international cooperation to make it possible.

Recently, one of the co-authors (J.-H. Ding) found an interesting *Vaccinium* in Chao’an Fenghuangshan Provincial Nature Reserve, Guangdong Province, China. It resembles *V.wrightii* A. Gray, a species endemic to Taiwan and the Ryukyu Islands, in having leaf-like floral bracts and white spherical-urceolate corollas (Li 1978; Yamazaki 1993; Li et al. 1998). However, after careful comparison of this plant to other similar species similar, we confirmed that it represents a species new to science and is here described and illustrated.

## ﻿Materials and methods

Flowering and fruiting materials were collected from Fenghuangshan, Chaozhou City, Guangdong Province, China, during several field trips from 2022 to 2023. The description was based on dried herbarium specimens. The online specimen photos (including type specimens) of *V.wrightii* in GH, K, and W were consulted, as well as physical herbarium specimens deposited at IBSC and PE. Measurements were obtained with a ruler, and small plant parts were observed and measured under a stereo microscope (Mshot-MZ101).

## ﻿Taxonomic treatment

### ﻿ Vaccinium chaozhouense

Taxon classificationPlantaeEricalesEricaceae

Y.H.Tong & J.H.Ding sp. nov.

ABDEC9D5-A22E-5ABA-B3CF-D8E7D3D4B3FA

urn:lsid:ipni.org:names:77332387-1

Fig. 1

#### Type.

China. Guangdong Province: Chaozhou City, Chao’an Fenghuangshan Provincial Nature Reserve, 980 m a.s.l., 10 May 2023 (fl.), *Yi-Hua Tong et al. TYH-2699* (holotype: IBSC, isotypes: KUN, PE).

#### Diagnosis.

The new species is most similar to *V.wrightii* A. Gray (including its variety with smaller habit and leaf blades, V.wrightiivar.formosanum (Hayata) H. L. Li) by having flowers with persistent and leaf-like bracts, and white spherical-urceolate corollas, but is distinguished by the presence of glandular trichomes on the abaxial surface of the leaf blade (vs. glabrous), shorter pedicels (4–6 mm vs. 5–15 mm), sparsely pilose (vs. glabrous) ridges of the corolla, and anther thecae longer than the tubules (vs. equal to or shorter than the tubules). A more detailed comparison of the two species is provided in Table 1.

**Table 1.** Morphological comparison of *Vacciniumchaozhouense* and *V.wrightii*. The data for *V.wrightii* are from Li (1978), Fang and Stevens (2005), and Yamazaki (1993) as well as the examination of the specimens listed in the text.

Characters	*V.chaozhouense*	*V.wrightii*
Abaxial surface of leaf blade	With evenly distributed glandular trichomes	Glabrous
Inflorescence length (cm)	1.5–4	5–8.5
Indumentum of inflorescence rachis	Pubescent or sometimes glabrous	Glabrous
Pedicel length (mm)	4–6	5–15
Indumentum of calyx	Pubescent or sometimes glabrous	Glabrous
Indumentum of abaxial surface of corolla	Sparsely pilose on edges, otherwise glabrous	Glabrous
Anther thecae	Longer than tubules	Equal to or shorter than tubules
Indumentum of disk	White-pubescent or sometimes nearly glabrous	Usually glabrous

**Figure 1.**
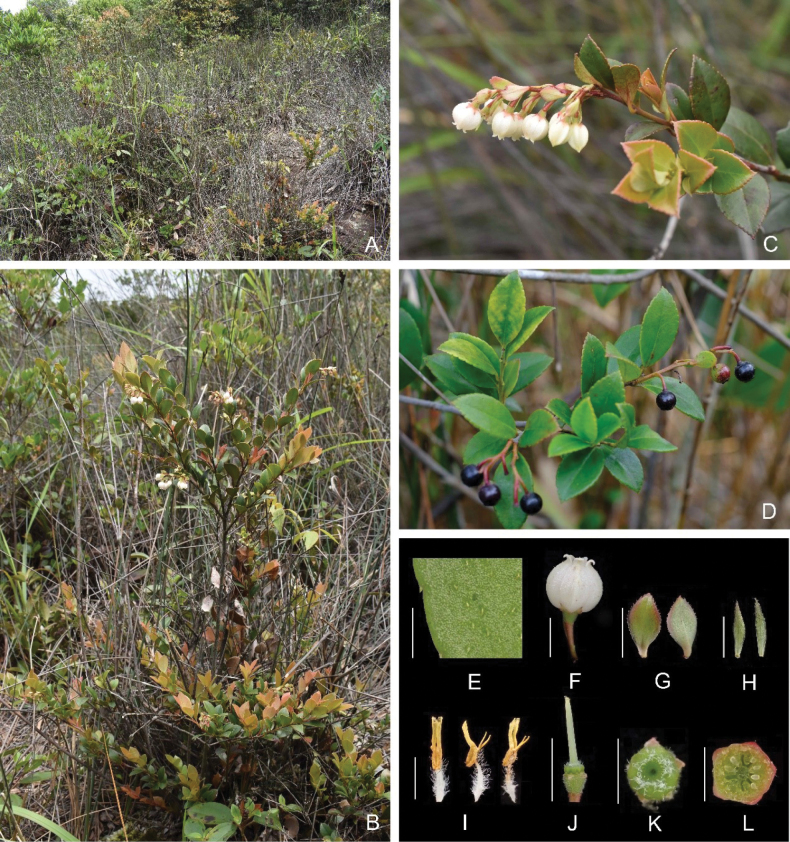
*Vacciniumchaozhouense***A** habitat **B** habit and flowering branchlets **C** flowering branch **D** fruiting branches **E** abaxial surface of leaf blade showing glandular trichomes **F** flower **G** bracts, adaxial (left) and abaxial (right) surfaces **H** bracteoles, adaxial (left) and abaxial (right) surfaces **I** stamens, ventral (left), lateral (middle) and dorsal (right) view **J** hypanthium (with calyx lobes removed), showing disk and style **K** disk, top view **L** ovary cross-section, showing the pseudo-10-locular ovary. Scale bars: 5 mm (**F–G, J**); 3 mm (**H**); 2 mm (**I, L**); 1.5 mm (**E, K**). Photographs by Yi-Hua Tong, except **D** by Jian-Hong Ding.

#### Description.

Evergreen shrubs, 0.3–1.5 m tall. Young branchlets densely pubescent, glabrescent. Leaves dense, spirally alternate; blades leathery or thickly leathery, elliptic or ovate, 1.5–3.1 × 0.7–1.8 cm, apex acute, base cuneate to broad cuneate, margin serrate, each serra tipped with a gland, abaxially with evenly distributed glandular trichomes throughout, adaxially glabrous, midvein raised abaxially, slightly impressed adaxially, lateral veins 4–7 on each side, slightly conspicuous abaxially, inconspicuous adaxially; petiole 2–3 mm long, initially pubescent, glabrescent at maturity. Racemes pseudoterminal and axillary, 1.5–4 cm long, with 3–7 flowers, rachis pubescent or sometimes glabrous; bracts persistent, leaf-like, ovate, elliptic or obovate, 3.5–8 × 2–4 mm, nearly glabrous on both sides, margin with (1–)5–15 glandular teeth per side, occasionally ciliolate at the apex; pedicel glabrous or sparsely pubescent on upper part, 4–6 mm long; bracteoles 2, caducous, usually borne at the middle and lower part (towards rachis) of the pedicel, occasionally at the upper part (towards hypanthium), lanceolate, 1.5–3 × 0.5–1 mm wide, glabrous on both sides, margin with (0–)1–2 glandular teeth, occasionally ciliolate at the apex. Hypanthium obconical, densely white-pubescent or sometimes glabrous; calyx limb 5-lobed to near base, calyx lobes triangular or broadly triangular, 1–1.5 × 1.2–1.5 mm, glabrous on both sides or sparsely pubescent abaxially, often with glandular teeth on the margin, apex acuminate, sometimes ciliolate. Corolla white, spherical-urceolate, slightly 5-ridged, 5–6 × 4–5 mm, sparsely pilose on the ridges abaxially, otherwise glabrous, pilose adaxially, apex shallowly lobed, lobes recurved, triangular, ca. 1.2 × 1.2 mm; stamens 10, 4.5–4.8 mm long, filaments 2–2.2 mm long, densely white-villous, anthers 2.5–2.7 mm long, thecae 1–1.2 mm long, tubules 1.5–1.7 mm long, spurs present, borne at the base of tubules, obliquely projected, ca. 0.8 mm long; style 4–5.5 mm long, stigma truncate, ovary pseudo-10-locular, each with several ovules, disk white-pubescent or sometimes nearly glabrous. Fruit globose, 4–4.5 mm in diameter, black when ripe; fruiting pedicel 4–6 mm long.

#### Etymology.

The species epithet is named after the type locality, Chaozhou City. The Chinese name is given as 潮州越橘 (Chinese pinyin: cháo zhōu yuè jú).

#### Distribution, habitat and conservation status.

This species is currently known only from the type locality, i.e., Fenghuangshan, the highest mountain in the Chaoshan district (an area of nearly 16,000 km^2^ in East Guangdong) with an elevation of 1497.8 m at the summit. *Vacciniumchaoanense* grows among shrubs on sunny volcanic rocks at an elevation of ca. 980 m. This kind of habitat where this species grows is actually a little unusual in Fenghuangshan, as most of Fenghuangshan area is covered with evergreen broadleaf forests. Only one population with < 30 individuals was found despite a careful search in the area. Thus, it is assigned a status of ‘Critically Endangered’ (CR, criterion D) following the IUCN Red List categories and criteria (IUCN 2012) and guidelines (IUCN Standards and Petitions Committee 2022). Because its distribution area is under the protection of Chao’an Fenghuangshan Provincial Nature Reserve, and it is not economically valuable, the threat risk seems low.

#### Phenology.

Flowering in May and fruiting in October–November.

#### Additional specimens examined.

*Vacciniumchaozhouense*: the same locality as the type, 18 May 2022 (fl), *Jian-Hong Ding s.n.* (IBSC); ibid., 31 October 2022 (fr.), *Jian-Hong Ding s.n.* (IBSC); ibid., 10 May 2023 (fl.), *Yi-Hua Tong et al. TYH-2700* (IBSC).

*Vacciniumwrightii*: Japan. Ryukyu: without precise locality, without date, *C. Wright 170* (holotype GH00015982, image, isotypes K000780593, image, NY00010772, image); without precise locality, 1887, *O. Warburg s.n.* (W, image); Gneka-Kesaji, 9 June 1930, *K. Kondo 1975* (PE00245894); Higashi-son, Gaji, in windy scrubs, 100 m a.s.l., 12 October 1990, *T. Yamazaki 6486* (PE00197326); Iriomote Island, Shirahama, on the seaside, 29 April 1980, *K. Inoue 1469* (IBSC0457605, PE00197336); Iriomote Island, on the way from Ootomi to Mt. Goza, in evergreen forest, 20–200 m a.s.l., 4 August 1981, *N. Fukuoka & M. Ito 180* (IBSC0457604); Iriomote Island, valley of Yuchin-gawa River, on rocky cliff at shady riverside, 6 April 2004, *K. Yonekura et al. 11346* (IBSC0741637); Iriomote Island, along Shirahama Forest Road, ca. 1 km from the entrance, on sunny slope around a marsh in a small valley, 120–130 m a.s.l., 3 April 2004, *Koji Yonekura 11213* (PE); Iriomote Island, in evergreen forest, 50 m a.s.l., 8 November 1985, *Toshiyuki Nakaike s.n.* (PE00197345); Nago-shi, NW slope of the Mt. Nago-dake, 200 m a.s.l., 11 March 1978, *J. Murata 4717* (PE00438051). CHINA. Taiwan: Hualien, Hsiaochingshui, 350 m a.s.l., 17 April 2002, *Tien-Tsai Chen 11872* (PE); Taipei, Taluntoushan, 13 June 1997, *Her-Long Chiang 496* (PE00197448).

#### Discussion.

*Vacciniumchaozhouense* can be assigned to V.sect.Bracteata Nakai according to Sleumer’s or Vander Kloet & Dickinson’s infrageneric classification system (Sleumer 1941; Vander Kloet and Dickinson 2009) by its terrestrial habit, pinninerved leaf blade with a serrate margin, persistent and leaf-like floral bracts, pseudo-10-locular ovary and black fruit, which match the characters of that section. Two other species are also similar to this new species in having the same leathery leaf texture, persistent, large, and leaf-like floral bracts, and similar urceolate or spherical-urceolate corolla shape, i.e., *V.eberhardtii* Dop from Vietnam and Thailand, which has also been recently reported from Guangxi, China (Tong et al. 2018), and *V.boninense* Nakai endemic to the Bonin Islands (also known as Ogasawara Islands). A key to the four species is provided below summarizing the morphological differences among them. Although these four species were assigned to V.sect.Bracteata for now, they bear unusual urceolate shape of corolla, while many other species in this section have tubular corolla. Meanwhile, a continuous coastal distribution from western Pacific Ocean islands to Indochina is presented by the four species. Thus, according to their morphological characteristics and distribution pattern, these four species may represent a distinct lineage in V.sect.Bracteata, and studies on their biogeography and speciation seem warranted.

### ﻿Key to *V.chaozhouense* and similar species

**Table:** 

1	Pedicel 2–4 mm; anther spurs oriented nearly straight upward, ca. 1.2 mm long, longer than half of anther tube; China (South Guangxi), Vietnam, and Thailand	***V.eberhardtii***
–	Pedicel 4–15 mm; anther spurs oriented obliquely upward, 0.3–0.8 mm long, shorter than or equal to half of anther tube	**2**
2	Leaf blade margin entire or nearly so, obscurely dentate; corolla urceolate; Japan (Bonin Islands)	***V.boninense***
–	Leaf blade margin evidently serrate; corolla spherical-urceolate	**3**
3	Abaxial surface of leaf blade with evenly distributed glandular trichomes; pedicels 4–6 mm long; corolla ridges sparsely pilose abaxially; anther thecae longer than tubules; China (East Guangdong)	***V.chaozhouense***
–	Abaxial surface of leaf blade glabrous; pedicels 5–15 mm long; corolla glabrous throughout abaxially; anther thecae equal to or shorter than tubules; China (Taiwan) and Japan (Ryukyu Islands)	***V.wrightii***

## Supplementary Material

XML Treatment for Vaccinium chaozhouense

## ﻿Additional information

### Author ORCIDs

Yi-Hua Tong https://orcid.org/0000-0002-5034-005X

### Data availability

All of the data that support the findings of this study are available in the main text.
